# Bilingual disadvantages are systematically compensated by bilingual advantages across tasks and populations

**DOI:** 10.1038/s41598-024-52417-5

**Published:** 2024-01-24

**Authors:** Vittoria Dentella, Camilla Masullo, Evelina Leivada

**Affiliations:** 1 Department of English and German Studies, Universitat Rovira i Virgili, Tarragona, Spain; 2 Department of Catalan Philology, Universitat Autònoma de Barcelona, Barcelona, Spain; 3 Institució Catalana de Recerca i Estudis Avançats (ICREA), Barcelona, Spain

**Keywords:** Psychology, Human behaviour

## Abstract

Bilingualism is linked to both enhanced and hampered performance in various cognitive measures, yet the extent to which these bilingual advantages and disadvantages co-occur is unclear. To address this gap, we perform a systematic review and two quantitative analyses. First, we analyze results from 39 studies, obtained through the PRISMA method. Less than 50% of the studies that show up as results for the term “bilingual disadvantage” report exclusively a disadvantage, that shows bilinguals performing worse than monolinguals in a task. A Bayesian analysis reveals robust evidence for bilingual effects, but no evidence for differences in the proportion of advantages and disadvantages, suggesting that when results from different cognitive domains such as executive functions and verbal fluency are analyzed together, bilingual effects amount to a zero-sum game. This finding was replicated by repeating the analysis, using the datasets of two recent meta-analyses. We propose that the equilibrium we observe between positive and negative outcomes may not be accidental. Contrary to widespread belief, advantageous and disadvantageous effects are not stand-alone outcomes in free variation. We reframe them as the connatural components of a dynamic trade-off, whereby enhanced performance in one cognitive measure is offset by an incurred cost in another domain.

## Introduction

One of the most heatedly debated topics in psycholinguistics concerns the effects of bilingualism on cognition. Many studies have linked bilingualism to an enhanced performance in tasks that tap into a range of executive function measures^1–3^. Although knowing multiple languages facilitates communication as well as exposure to a variety of cultures and is thus always an advantage^4^, the term “bilingual advantage” has a narrower meaning. It was introduced to refer specifically to results that show that bilinguals may perform better than monolinguals in certain cognitive tasks, mostly pertaining to conflict resolution^2,3,5^. In parallel to the line of literature that produced results showing that bilinguals demonstrate a superior performance in certain tasks, another line started adducing results that revealed a bilingual disadvantage, mainly in the domain of semantic fluency and naming^6,7^.

As the original finding of a bilingual advantage in executive functioning was put to test in an ever-expanding range of child and adult, neurotypical and neuroatypical populations, conflicting results started emerging, even when using the exact same tasks. Some studies found a robust bilingual advantage in executive functioning^3^, while others failed to find evidence for its existence, obtaining results that suggested that the effect is indistinguishable from zero^8,9^. This inconsistent occurrence of the bilingual advantage gave rise to concerns about its status as a robust phenomenon^10,11^. While several explanations have been offered for both sets of results^12–16^, the degree to which bilingual advantages and disadvantages co-occur is still unknown. This is an important knowledge gap, given that the field of bilingualism research has been repeatedly linked to meta-analyses that reveal publication bias against negative results^17,18^. Some studies have even claimed that the entire idea of a bilingual advantage may have stemmed from this publication bias^17^. Since some of the effects that attest to publication bias did not replicate^19,20^, a clearer understanding of the prevalence of bilingual advantages and disadvantages is missing.

Importantly, the problem runs deeper than not knowing the degree of co-occurrence of advantages and disadvantages in bilingual cognition, because most studies describe *either* advantages *or* disadvantages, as if they were stand-alone effects. What is consistently left in the margins is the appreciation of the fact that enhancing one aspect of a system (e.g., any goal-directed system, including cognition) entails a cost for another aspect of the same system^21^. Succinctly put, enhanced computational performance never comes for free^21^. Since human cognition is no exception to this rule, we expect enhancements (caused by any trigger, not just bilingualism) to be counterbalanced by disadvantages. This means that if a(n) (dis)advantageous effect is found, it likely forms part of a *trade-off*, because enhanced performance in one domain is bound to be compensated in another domain. In biology, the notion of the trade-off refers to a negative correlation between processes that make use of the same finite resources within an organism^22^. From an evolutionary point of view, such trade-offs are frequent across species and emerge because one trait cannot be optimized without creating an expense for other traits, given that organisms function together as *integrated wholes* in the Darwinian sense^23^. From a developmental point of view, trade-offs often translate into a negative relationship between traits, based on morphological, physiological, and environmental characteristics that contribute to the development of an organism (e.g., a speed vs. stamina trade-off in Olympic sprinters vs. marathoners^24^).

Although a few studies have mentioned the possibility that specific advantages and disadvantages share origin (e.g., Luo et al.^25^ ask whether it is possible that a common mechanism underlies the opposite effects that they found for letter and category fluency in bilinguals), only a handful of them have approached the effects of bilingualism on cognition through explicitly proposing a trade-off that links them together. Struys et al.^26^ present evidence for a speed-accuracy trade-off in executive functioning, with the effect being specific to the bilingual populations they tested. More specifically, they report null results in terms of a bilingual advantage and clear-cut results for speed-accuracy trade-offs in bilinguals, but not in monolinguals: bilinguals either boost their response times sacrificing accuracy or they prioritize accuracy, slowing down their performance^26,27^. Marsh et al.^28^ observe that decreased semantic fluency in bilinguals can be explained as an offset against enhanced executive functions or metalinguistic awareness. Last, Leivada et al.^29^ find evidence for a speed-accuracy trade-off in the grammatical domain. Testing the fallibility of monolinguals and bilinguals in grammatical illusions (i.e., sentences that appear meaningful and well-formed, but are not), bilinguals were found to be better than monolinguals in detecting the grammatical anomalies in the seemingly well-formed sentences, but they were also slower in providing an answer. Leivada et al.^29^ frame this finding through proposing the Plurilingual Adaptive Trade-off Hypothesis, according to which, the bilinguals’ adaptive alteration of their language control abilities^30^ may result in enhanced (pragmatic) monitoring, but this advantage is part of a larger bundle of effects that are not all advantageous.

The trade-off approach carries important implications for the findings of many (meta-)analyses, and especially for the ones that report both a null finding and a negative finding (i.e., bilingual disadvantage), or group together the two, eventually juxtaposing this “negative/null” category to the category of positive findings (i.e., bilingual advantage). For example, de Bruin et al.^17^ examine whether the publication of conference abstracts is affected by the stance they take in the bilingual-advantage debate. They find evidence for a publication bias: studies that report a disadvantage are the least likely to be published, while studies supporting the bilingual-advantage hypothesis are the most likely to be published. The classification system they employ consists of 4 mutually exclusive categories: (i) positive result (i.e., evidence in favor of a bilingual advantage), (ii) mixed result, predominantly positive (i.e., evidence in favor of a bilingual advantage, albeit not in all tasks or populations), (iii) mixed result, predominantly negative (i.e., partial/inconsistent evidence in favor of a bilingual advantage, but failure to find it in conditions where it was expected), and (iv) negative or null result (i.e., evidence in favor of a bilingual disadvantage or absence of significant differences between monolinguals and bilinguals). The last category groups together two very different outcomes: an effect that has been argued to exist and an effect that has been argued to be indistinguishable from zero. However, under the perspective we have laid out so far, finding a bilingual disadvantage is potentially interpreted as indirect evidence for a bilingual advantage in another domain. Consequently, treating a negative result as synonymous to a null result, and putting them together in one category, does not do justice to the correlation between advantages and disadvantages^20^.

To give a second example of how bringing trade-offs into the picture is informative in relation to interpreting the conclusions of meta-analyses, Lehtonen et al.^18^ argue that their analysis of 152 studies does not provide systematic support for the view that bilingualism grants an advantage in cognitive control functions in adults. Moreover, they report finding evidence for a small bilingual disadvantage in verbal fluency tasks. Yet if advantages and disadvantages are viewed as two sides of the same coin, finding a disadvantage probably entails the presence of an advantage (unless of course one explains why bilingualism would behave in a way that challenges current knowledge about the workings of human cognition^21,^ by virtue of causing enhancements that are not compensated for). If enhanced computational performance indeed does not come for free, observing a cost in the form of a disadvantage inevitably raises the question of what enhancement this disadvantage is compensating for. From this perspective, Lehtonen et al.^18^ provide direct evidence for a bilingual disadvantage and indirect evidence for a bilingual advantage. This reframing of their results seems to be at odds with their conclusion that bilingualism is not *reliably* associated with cognitive benefits.

Although it remains an open question whether such advantages should be attributed to monitoring two language systems, and by extension to bilingualism per se, as opposed to the interaction of bilingualism with other factors (e.g., socio-economic status, cultural background^9,31,32^, our focus is not on the much-discussed, yet still unclear, origin of the observed effects (see^33^ for a discussion of open questions in that domain), but on their significantly understudied distribution. In a nutshell, the purpose of this study is to determine the extent to which advantageous and disadvantageous effects complement each other across different child and adult, neurotypical and neuroatypical populations. Subsequently, we discuss the nature of the cognitive trade-offs, specifically as they emerge in the context of bilingualism research and present their main characteristics in terms of development (i.e., how they come to exist in the individual) and plasticity (i.e., how they evolve over time in the individual).

## Method

We first performed a systematic review and a quantitative analysis of the literature on the bilingual disadvantage. The reason for choosing the bilingual disadvantage as the starting point has to do with concerns about publication biases. Given that evidence for a publication bias against negative results has been reported in the literature^17,18^, the starting point need be the least plentiful category of studies, examined against the most plentiful category in order to determine the degree to which the two effects co-exist within and across different populations.

The review was conducted according to the PRISMA Statement^34^. A systematic search of the literature was conducted in the following databases: PsycInfo, PsycExtra, PsycBooks, APA Journals, and PubMed. The search strategy consisted of the following keywords: “bilingual” AND “disadvantage”. The searches were conducted in March 2021. A total of 150 articles were obtained from this search procedure. Subsequently, duplicates were removed, and the remaining abstracts were screened for content.

**Figure 1:**
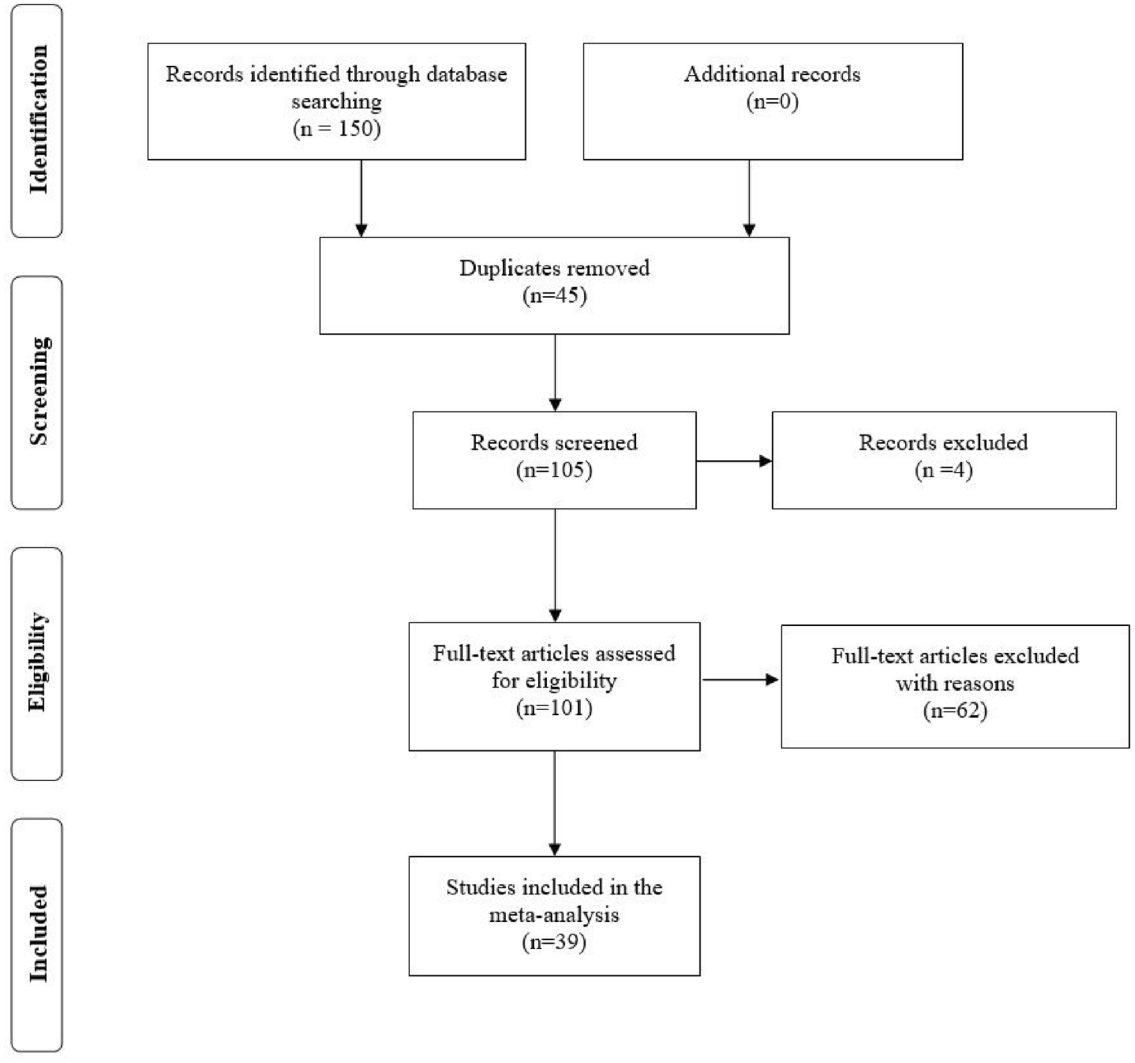
PRISMA flow chart.

The selection of relevant studies was conducted based on previously determined inclusion and exclusion criteria. First, studies had to present original experimental results that were obtained from testing behavioral outcomes (e.g., executive functions, fluency, speech patterns recognition, metalinguistic knowledge, etc.). Therefore, meta-analyses, review articles, and theoretical articles were excluded. Second, studies had to be written in English. Third, studies had to be published after 1950. Fourth, data from at least one monolingual and one bilingual group had to be reported. Last, screened abstracts that outlined finding only advantages, without any acknowledgment of behavioral testing for possible disadvantages or some reference to specific factors that may weaken the expected advantage, were excluded (e.g., ethnographic studies that talked about a bilingual advantage in the context of individual interviews were excluded). If the abstract was not informative enough, the full text was screened. The obtained database covers results from 39 studies, 60 experiments, and 7,830 participants. Figure 1 presents the screening and selection process.

The studies are classified into the following five categories:

(i) Advantage & Disadvantage: Studies that report both an advantage and a disadvantage.
(ii) Neither Advantage, Nor Disadvantage: Studies that report neither an advantage, nor a disadvantage. Language group effects are either absent or, when present, are attributed to factors external to bilingualism.
(iii) Advantage: Studies that report only an advantage.
(iv) Disadvantage & External Advantage: Studies that report a disadvantage for a population/language group for which an advantage has also been reported in the literature (i.e., the second phase complementary studies). In the second phase, 18 complementary studies were selected and examined (following the process described below) with the aim to synthesize the overall characteristics of the populations identified in the first phase in terms of global advantages and disadvantages.
(v) Disadvantage: Studies that report a disadvantage for a population/language group for which no advantage has been found in the literature.

In the Supplementary Information file, Supplementary Table 1 presents a summary of the results, and Supplementary Table 2 presents the demographics of our sample, split in three age groups, together with their distribution in terms of results. The pool of data, the complete list of studies that were analyzed for this review, and an individual analysis of each study are available at https://osf.io/8w6ux/.

Encompassing a variety of tasks and populations, our pool of data suggests that about 30% of the screened studies either report both an advantage and a disadvantage [category (i)] or only an advantage [category (iii)]. Excluding category (ii), which reports no bilingual effects and amounts to about 30% of the results, the remaining 40% [n = 16, category (iv)] of studies report only a bilingual disadvantage.

In the second phase, we performed a targeted search of the literature to determine to what extent bilingual disadvantages and advantages co-occur across populations. The bilingual groups in this complementary pool of data were individually matched to the “disadvantage-only” studies in the PRISMA-obtained pool of data for age, type of bilingualism, and linguistic profile. This additional database covers results from 26 experiments and 3,448 participants. We determined that all the populations for which only disadvantages have been found in the first pool of data have been linked to bilingual advantages in other studies. The full list of complementary studies, matched to the studies obtained through the PRISMA protocol, is given and discussed at https://osf.io/8w6ux/.

One important note is due to the compilation of the list of complementary studies. To bring them together, we performed an extensive literature review (without using the PRISMA protocol as each search was tailored to one study in the original pool of data), and we identified those studies that come closest to the original study in terms of as many of the following characteristics as possible: the languages used (spoken or signed), the tested age groups, and the overall linguistic profile. However, as in all meta-analyses, the obtained comparisons are at best approximate. We cannot provide a perfect or close-to-perfect match across all variables, because such a degree of matching is not possible for many of the tested populations. This point relates to the visibility of certain groups in bilingualism research: the level of approximation is solid when one compares studies which target populations that have been thoroughly tested (e.g., bilingual college students of English and Spanish in the US), but less solid when one has very few studies from which a close match can be drawn, because the target populations use severely understudied minority, non-standard, or non-official languages. In terms of our methods, faced with the choice of favoring either solid matching (by including only those studies that deal with populations that have a high visibility and can afford the firmest comparisons) or inclusion (by including those studies that can only partially be matched for a subset of their characteristics), we chose the latter. However, we highlight again the caveat of comparability: there is no perfect match between the groups and all comparisons operate on some level of approximation that is heavily influenced by the overall visibility and representation of certain groups.

Turning to the quantitative analysis of the present study, this differs from previous meta-analyses on the topic in many critical respects. First, it does not focus exclusively on effects found in the domain of executive functions. If one examines the recent meta-analyses of bilingual adaptations, it is likely that one will obtain an incomplete picture, because these analyses involve samples that represent only the domain of executive control^12,18,19,35,36^. However, bilingualism may have an impact on a variety of cognitive measures (e.g., syntactic processing, metalinguistic awareness, word learning). There is no reason for confining our analyses to results that come from one cognitive domain, when there is ample evidence for bilingual adaptations across domains. To remedy this bias in the representation of bilingual adaptations and provide a more complete picture, no cognitive domain was excluded from our pool of data. Second, the process of outlier removal in meta-analyses may contribute to masking group differences between monolinguals and bilinguals^19^, leading to a null effect. To bypass this challenge, the present study will use an alternative quantitative approach, following Grundy^19^. Last, unlike previous meta-analyses and quantitative analyses on the topic, this analysis does not take bilingual advantages and disadvantages as unique outcomes within a study. In other words, if a study finds evidence for both advantages and disadvantages, both results are represented in our analysis.

Our quantitative analysis has two aims. The first aim is to determine whether studies in our pool of data are more likely to favor a null outcome or find evidence for bilingual effects. To measure this, we adapt the quantitative approach of Grundy^19^ in terms of coding, while using the same statistical analyses: Studies were coded as 1 if they found evidence for a bilingual advantage or disadvantage, and as -1 if no group differences were reported. If a study found both types of effects (e.g., a bilingual advantage in reaction times and a bilingual disadvantage in accuracy), this was coded as two entries, one for each effect.

The second aim is to zoom into the studies that find evidence for bilingual effects in order to determine the overall proportion of bilingual advantages and disadvantages. To this end, studies that found evidence for bilingual adaptations were re-classified in terms of their type of findings. If a study found evidence for a bilingual advantage, it was coded as 1. If a study found evidence for a bilingual disadvantage, it was coded as − 1. If a study found evidence for both effects, two entries were created for this study, following the previously described process.

Critics of the bilingual-advantage hypothesis have proposed that a Bayesian approach is more appropriate when one seeks to examine the effects of bilingualism on executive functions^37^. Although the present quantitative analysis does not focus exclusively on executive functions, we concur about the importance of running analyses that are informative about how likely the data are to occur under the null vs. the alternative hypothesis. For this reason, Bayesian analyses are reported. The analyses were run using jamovi, version 2.2^38^. Table 1 represents the input, which corresponds to the studies obtained through the PRISMA protocol (Fig. 1). Assessing for possible sample biases, 5 studies from the pool of data were excluded from the quantitative analysis because they involved very small sample sizes (< 30), which have been argued to provide a blurry picture in bilingualism research^39^, carrying the risk of a substantial increase of the rate of Type I errors^10^.

**Table 1:** Data for the studies included in the quantitative analysis (references at https://osf.io/8w6ux/).

Study	N	Effect (− 1 = no difference, 1 = B > M, 1 = M > B)	Effect (advantage, disadvantage, null)
Baus et al. (2020)	53	1	Disadvantage
Broos et al. (2018) exp. 1	123	− 1	Null
Broos et al. (2018) exp. 2	123	− 1	Null
Broos et al. (2018) exp. 3	123	− 1	Null
Broos et al. (2018) exp. 4	108	− 1	Null
Broos et al. (2018) exp. 5	108	− 1	Null
Broos et al. (2018) exp. 6	108	− 1	Null
Chen et al. (2013) exp. 1	337	1	Disadvantage
Chen et al. (2013) exp. 2	62	1	Disadvantage
Chen et al. (2013) exp. 3	90	1	Disadvantage
Coderre et al. (2013) exp. 1	60	1	Advantage
Coderre et al. (2013) exp. 2	60	1	Advantage
Coderre et al. (2013) exp. 3	60	1	Advantage
Desjardins et al. (2019)	61	1	Disadvantage
Desjardins et al. (2019)	61	1	Advantage
Filippi et al. (2020)	330	− 1	Null
Folke et al. (2016) exp. 1	62	1	Disadvantage
Folke et al. (2016) exp. 1	62	1	Advantage
Francis and Baca (2014)	216	1	Disadvantage
Kormi-Nouri et al. (2012)	1600	1	Disadvantage
Kormi-Nouri et al. (2012)	1600	1	Advantage
Kousaie et al. (2014)	218	− 1	Null
Lam and Sheng (2020)	106	1	Disadvantage
Lam and Sheng (2020)	106	1	Advantage
Lange-Kuettner et al. (2017) exp. 2	81	1	Advantage
Li et al. (2017)	96	1	Disadvantage
Marsh et al. (2019)	197	1	Advantage
Marton et al. (2017)	77	1	Advantage
Meir and Armon-Lotem (2017)	120	1	Disadvantage
Misdraji-Hammond et al. (2015)	126	1	Disadvantage
Morini and Newman (2020) exp. 1	64	1	Disadvantage
Morini and Newman (2020) exp. 2	64	− 1	Null
Morini (2014) exp. 1	64	1	Disadvantage
Morini (2014) exp. 1	64	1	Advantage
Morini (2014) exp. 2	64	1	Advantage
Paap and Greenberg (2013) exp. 1	80	− 1	Null
Paap and Greenberg (2013) exp. 2	86	− 1	Null
Paap and Greenberg (2013) exp. 3	107	− 1	Null
Paap et al. (2017)	230	− 1	Null
Pyers et al. (2009)	55	1	Disadvantage
Regalado et al. (2019)	60	− 1	Null
Runnqvist et al. (2013)	109	1	Disadvantage
Sadat et al. (2012) exp. 1	70	1	Disadvantage
Sadat et al. (2012) exp. 2	70	1	Disadvantage
Sadat et al. (2016)	90	1	Disadvantage
Sadat et al. (2016)	90	1	Advantage
Sandoval et al. (2010) exp. 1	99	1	Disadvantage
Sandoval et al. (2010) exp. 2	99	1	Disadvantage
Schmidtke (2014)	53	− 1	Null
Schmidtke (2016)	101	1	Disadvantage
Schulz and Grimm (2019) exp. 1	160	1	Disadvantage
Schulz and Grimm (2019) exp. 2	160	1	Disadvantage
Sheppard et al. (2016)	215	− 1	Null
Tao et al. (2015)	220	1	Disadvantage
Tao et al. (2015)	220	1	Advantage
Verhoeven et al. (2011)	1108	1	Disadvantage
J. Wu et al. (2019)	102	1	Advantage

## Results

### PRISMA-obtained pool of data: overall effects

**Figure 2:**
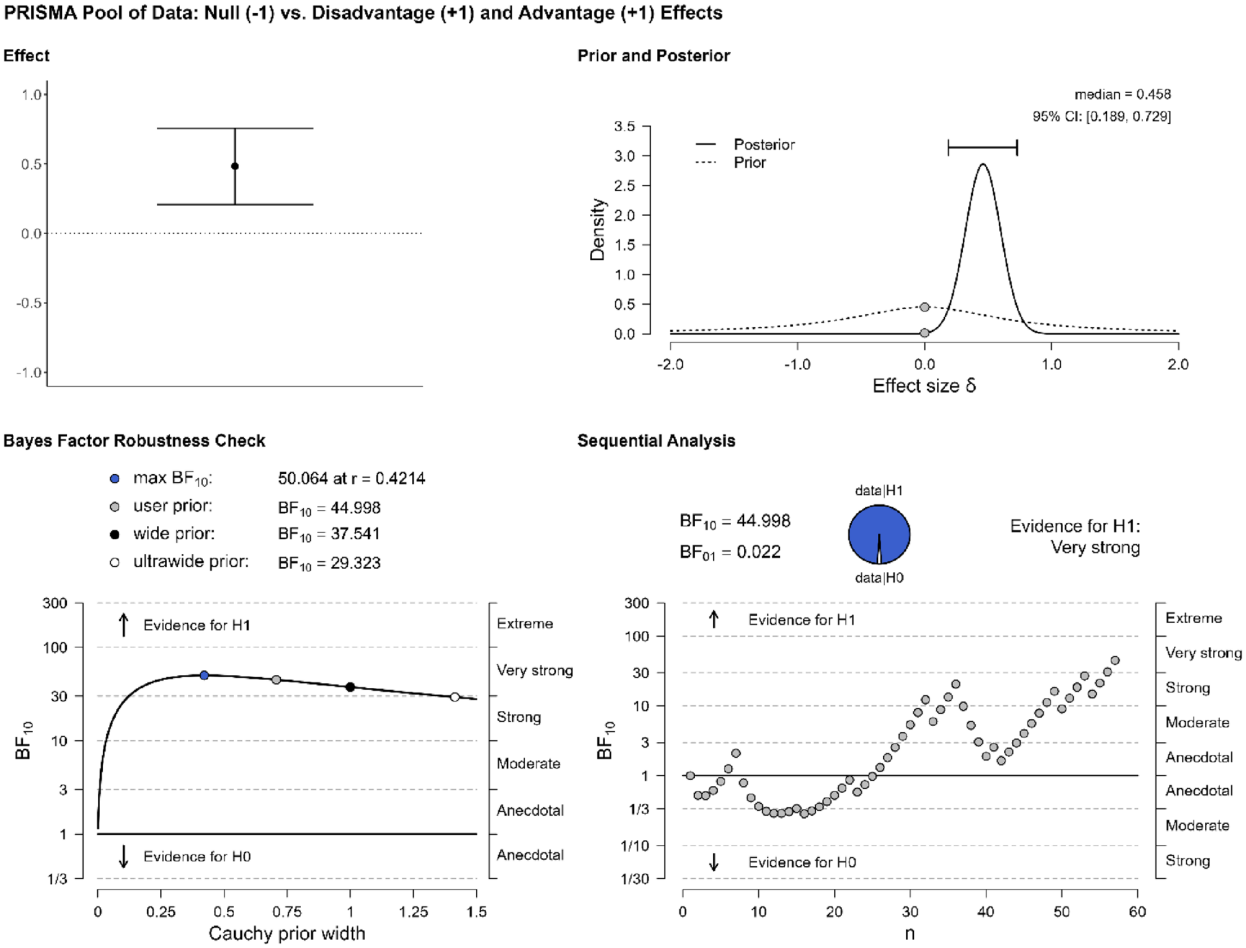
Evidence for bilingual adaptations in the pool of data.

The first analysis concerns the overall effects: Do studies in our pool of data provide evidence for bilingual adaptations? 34 studies were included in this analysis (Table 1). A Bayesian one-sample t-test suggests that the answer is positive, BF_10_ = 44.998. Details about the interpretation of the Bayes factor (BF) are given in the Supplementary Information. As Fig. 2 shows, the evidence in favor of interpreting the data as more likely under the alternative hypothesis (i.e., the hypothesis that studies in our pool of data are more likely to find evidence for bilingual effects than null effects) is very strong. In the panel “Effect”, we see the overall pattern across studies reporting a bilingual effect (1) or not (− 1). The circle represents the mean across studies and the error bars represent standard error. In the panel “Prior and Posterior”, the prior shows the starting probability before introducing the data, and the posterior represents the “updated” probability, after the data has been factored in. The panel “Bayes Factor Robustness Check” shows how the BF would change if different priors had been chosen. In our case, the BF is stable across various prior specifications, indicating the robustness of our findings. The sequential analysis represents the progression of the BF as each new study enters the analysis. This panel shows that the obtained evidence is very strongly in favor of the alternative hypothesis, which supports the existence of bilingual effects (taking positive and negative outcomes together) as opposed to null outcomes.

### PRISMA-obtained pool of data: type of effects

**Figure 3:**
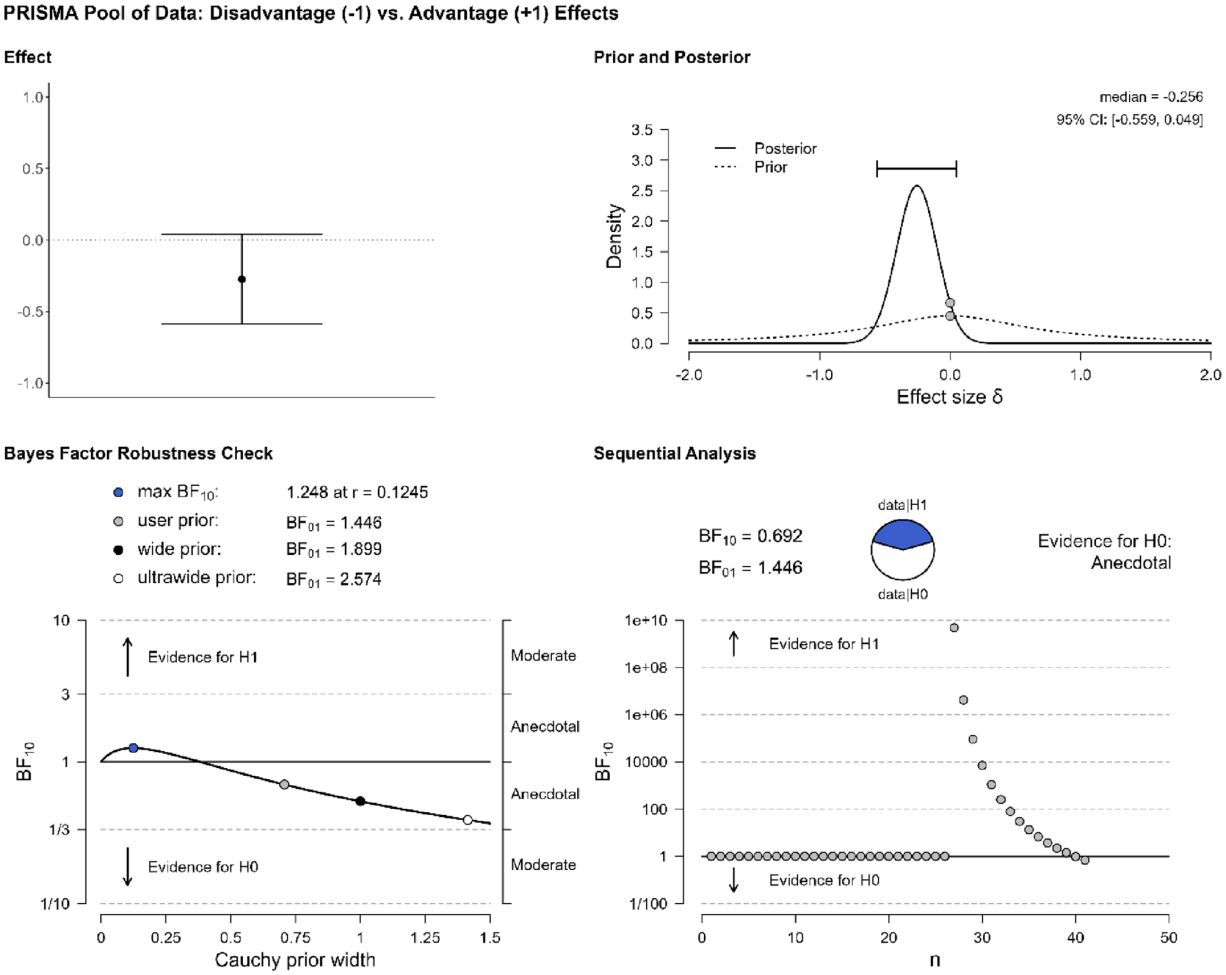
Evidence for the type of effect (advantage vs. disadvantage) in the pool of data.

The first step of our quantitative analysis has provided evidence for bilingual adaptations. The second step is to determine whether one type of effects, advantages or disadvantages, is more frequently attested, by looking into the overall proportion of bilingual advantages and disadvantages. The literature suggests that publication biases affect the publication of negative results^17,18^. The prediction, based on the assumption that the category of bilingual advantages is far more plentiful than that of bilingual disadvantages, is that this analysis will reveal that the data are more probable under the alternative hypothesis (i.e., that one effect is more frequently attested than the other). Repeating the previous analysis while focusing only on the studies in Table 1 that find bilingual effects (n = 26), a Bayesian one-sample t-test suggests that there is no evidence that one hypothesis is more strongly supported than the other, BF_10_ = 0.692. BFs between 0.33 and 3 are considered as anecdotal or inconclusive, not offering robust evidence for either hypothesis^40^. Since the data are equally likely under either the null or the alternative hypothesis, we cannot conclusively say that either advantages or disadvantages are more prevalent in our pool of data. Figure 3 shows the distribution of the results, marking the evidence for the null hypothesis as anecdotal.

Notice that in this analysis we have only included studies that find evidence for bilingual effects, excluding those that find a null result. The reason is conceptual: If a study fails to find evidence for bilingual adaptations, it cannot be determined whether this happens because no real-world effects existed in the first place or because the employed tasks, testing conditions, or analyses were not sensitive enough to pick up on the effects. Consequently, it is possible that studies with a null outcome affect our sample, by virtue of being included in the analyses as potential hosts of bilingual adaptations, when their status as such cannot be conclusively determined. However, to provide the full picture, we reran the previous analysis including the studies that find a null outcome (n = 8). We follow Grundy^19^ in coding null outcomes with 0, advantages with 1, and disadvantages with − 1. Again, the results of a Bayesian one-sample t-test provide only anecdotal evidence for the null (BF_10_ = 0.602).

**Figure 4:**
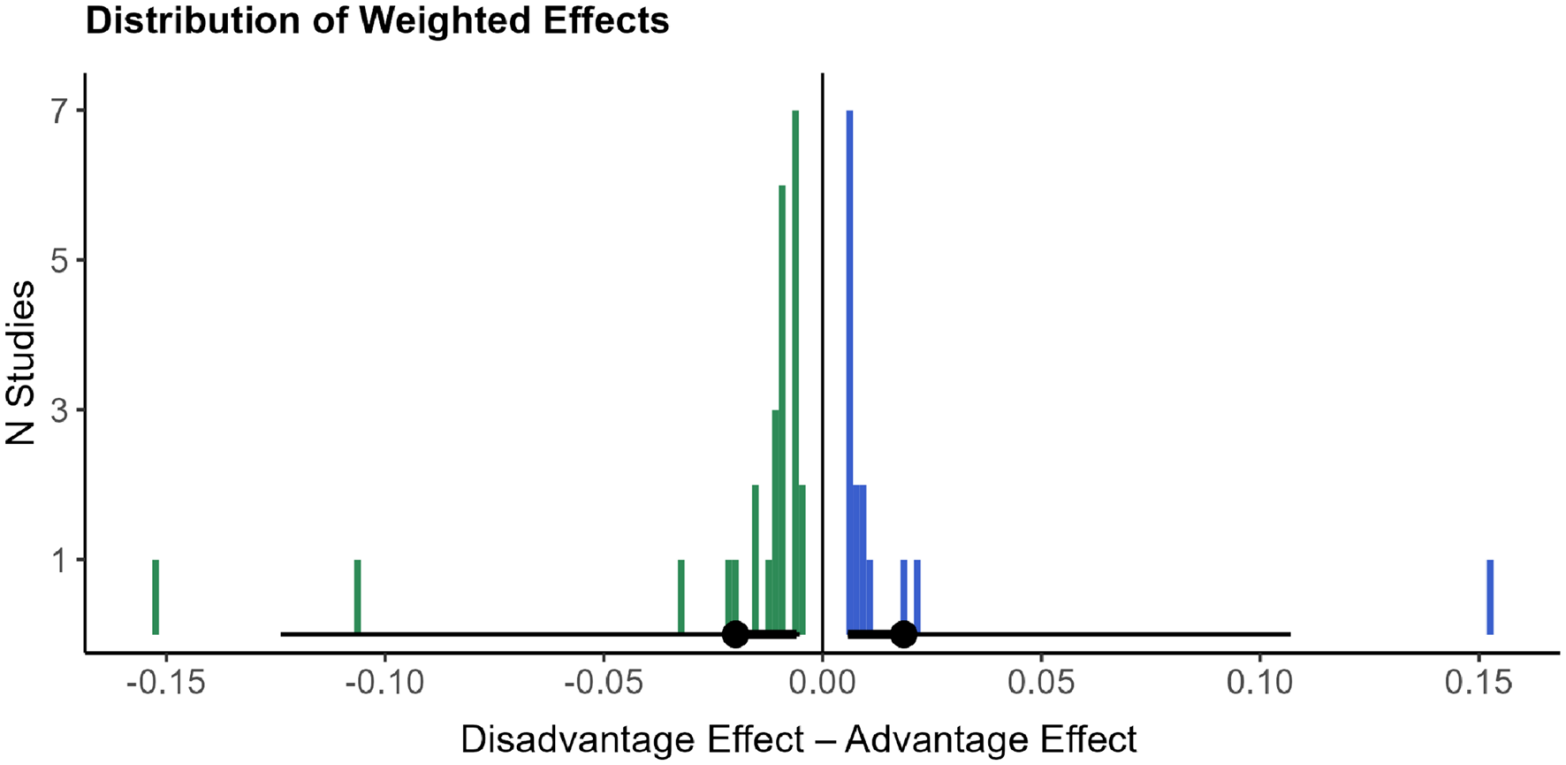
Weighted performance.

As Grundy^19^ notes, one of the main concerns about vote-counting analyses like the one we employed is that the data may not be weighted fairly if sample size is not considered. The issue of sample and effect sizes is particularly relevant in the literature that discusses bilingual adaptations^10,37^. To alleviate such concerns, we followed the weighting procedure of Grundy^19^: A sample size correction was applied to the data (the n = 39 studies originally obtained through the PRISMA method) before re-running the analyses. Each study was assigned a weight by dividing the study’s sample size by the total sample size of all studies in our pool of data. This proportional weight was then multiplied by the effect score (i.e., − 1 for a bilingual disadvantage, 1 for a bilingual advantage) to yield a weighted score. Using this approach, a Bayesian one-sample t-test showed moderate evidence in favor of interpreting the data as more probable under the null hypothesis (i.e., equal overall proportion of bilingual advantages and disadvantages), BF_10_ = 0.222. Figure 4 shows the distribution of the weighted performance.

### L&D pool of data: analyses replication

One caveat of our quantitative analysis is that we cannot discard the possibility that our search terms, which focused on bilingual disadvantages, may have biased our main findings. Succinctly put, it is possible that our finding that bilingual advantages and disadvantages are equally prevalent in our pool of data is an artifact of our selection process. Consequently, this result could possibly not be sustained if we analyzed a different pool of data, which uses different search terms and inclusion criteria. To address this caveat, we replicate the main finding by rerunning the previous analyses using a second pool of data. This second pool of data contains the datasets of two recent meta-analyses: Lehtonen et al.^18^ and Donnelly et al.^36^. The former used the search terms “bilingual” and “monolingual” and terms referring to various domains of cognitive control and executive functioning. The latter was based on searches that involved a combination of “bilingual” or “bilingualism” with “executive control”, “executive function”, “inhibition”, or “interference control”. Together the two datasets provide a second pool of data (henceforth, the L&D pool of data) that consists of 186 studies which were analyzed also in Grundy^19^ and are here taken as starting point. For our analyses, 7 studies were removed to avoid double representation, as they were already included in our original pool of data. 12 studies were removed because the full text was not available. 5 studies were removed because they were not in English. 3 studies were removed as they did not report bilingual/monolingual comparisons. 1 study was removed as it did not report the number of participants. 53 studies were removed for having very small bilingual samples (6–29 participants; cf.^10,39^ on the consequences of small sample biases). Τhe remaining studies (n = 105) were coded and analyzed following the same procedure presented above for the first pool of data. Table 2 represents the input, which corresponds to the studies that form the L&D pool of data.

**Table 2:** Data for the studies included in the quantitative analysis (L&D pool of data) (references at https://osf.io/8w6ux/).

Study	N	Effect (− 1 = no difference, 1 = B > M, 1 = M > B)	Effect (advantage, disadvantage, null)
Antón et al. (2014)	360	− 1	Null
Antoniou et al. (2016)	136	1	Advantage
Bak et al. (2014) Exp. 1	60	1	Advantage
Batres (2013) Study 1	96	1	Disadvantage
Batres (2013) Study 2	96	1	Disadvantage
Bialystok (2006)	97	1	Advantage
Bialystok et al. (2004) Study 2	94	1	Advantage
Bialystok et al. (2005) Study 3	96	− 1	Null
Bialystok et al. (2008a) Study 2	66	1	Disadvantage
Bialystok et al. (2008a) Study 2	66	1	Advantage
Bialystok et al. (2008b)	96	1	Disadvantage
Bialystok et al. (2008b)	96	1	Advantage
Bialystok et al. (2014) Study 1	130	1	Disadvantage
Bialystok et al. (2014) Study 1	130	1	Advantage
Bialystok et al. (2014) Study 2	108	1	Disadvantage
Bialystok et al. (2014) Study 2	108	1	Advantage
Bialystok et al. (2017)	168	1	Disadvantage
Bialystok et al. (2017)	168	1	Advantage
Bice and Kroll (2015)	69	1	Advantage
Billig and Scholl (2011)	83	1	Disadvantage
Billig and Scholl (2011)	83	1	Advantage
Blumenfeld and Marian (2011)	60	1	Advantage
Blumenfeld and Marian (2013)	61	1	Advantage
Blumenfeld and Adams (2014) Exp. 2	120	− 1	Null
Blumenfeld and Adams (2014) Exp. 1	60	1	Advantage
Blumenfeld et al. (2016)	60	1	Advantage
Bogulski et al. (2015)	79	1	Advantage
Bonifacci et al. (2011)	68	1	Advantage
Brito et al. (2016)	100	1	Advantage
Brown (2015)	71	1	Disadvantage
Calvo and Bialystok (2014)	175	1	Disadvantage
Calvo and Bialystok (2014)	175	1	Advantage
Coderre and van Heuven (2014)	76	1	Advantage
Costa et al. (2008)	200	1	Advantage
Costa et al. (2009) Exp. 1	120	− 1	Null
Costa et al. (2009) Exp. 2	124	1	Advantage
Craik and Bialystok (2006)	60	1	Disadvantage
Craik and Bialystok (2006)	60	1	Advantage
de Bruin et al. (2015)	76	− 1	Null
Delcenserie and Genesee (2017)	60	1	Advantage
Duñabeitia et al. (2014) Exp. 1	504	− 1	Null
Duñabeitia et al. (2014) Exp. 2	504	− 1	Null
Emmorey et al. (2008)	45	1	Advantage
Engel de Abreu et al. (2012)	80	1	Advantage
Feng (2008) Study 3	94	1	Disadvantage
Feng (2008) Study 3	94	1	Advantage
Fernandes et al. (2007)	104	1	Disadvantage
Friesen et al. (2015)	165	1	Disadvantage
Friesen et al. (2015)	165	1	Advantage
Gathercole et al. (2014) Exp. 1	650	1	Disadvantage
Gathercole et al. (2014) Exp. 1	650	1	Advantage
Gathercole et al. (2014) Exp. 2	557	1	Disadvantage
Gathercole et al. (2014) Exp. 2	557	1	Advantage
Gathercole et al. (2014) Exp. 3	354	1	Advantage
Gold et al. (2013) Exp. 2	80	1	Advantage
Gollan et al. (2002)	60	1	Disadvantage
Greene (2015) Exp. 1	180	− 1	Null
Greene (2015) Exp. 2	164	− 1	Null
Grundy et al. (2017) Study 1	59	1	Advantage
Grundy et al. (2017) Study 2	111	1	Disadvantage
Grundy et al. (2017) Study 2	111	1	Advantage
Grundy et al. (2017) Study 3	111	1	Disadvantage
Grundy et al. (2017) Study 3	111	1	Advantage
Guido Mendes (2015)	115	1	Disadvantage
Guido Mendes (2015)	115	1	Advantage
Gutierrez (2009) Exp. 1	145	− 1	Null
Gutierrez (2009) Exp. 2	145	− 1	Null
Gutierrez (2009) Exp. 3	145	− 1	Null
Gutierrez (2013)	240	1	Advantage
Heidlmayr et al. (2014)	64	1	Advantage
Hermans (2012)	110	1	Disadvantage
Hermans (2012)	110	1	Advantage
Hernández et al. (2013) Exp. 1	174	1	Advantage
Hernández et al. (2013) Exp. 3	77	− 1	Null
Hernández et al. (2010) Exp. 1	82	1	Advantage
Houtzager et al. (2017)	100	1	Advantage
Incera and McLennan (2016)	60	1	Disadvantage
Incera and McLennan (2016)	60	1	Advantage
Incera (2016)	180	1	Disadvantage
Incera (2016)	180	1	Advantage
Jiao et al. (2017) Exp. 1	58	1	Advantage
Jiao et al. (2017) Exp. 2	58	1	Advantage
Johns et al. (2016)	86	1	Disadvantage
Kapa and Colombo (2013)	79	1	Disadvantage
Kapa and Colombo (2013)	79	1	Advantage
Kaushanskaya and Marian (2009)	60	1	Advantage
Kazemeini and Fadardi (2016)	60	1	Advantage
Keijzer (2013)	173	1	Disadvantage
Keijzer (2013)	173	1	Advantage
Kerrigan et al. (2017)	60	1	Advantage
Kirk et al. (2014)	80	− 1	Null
Kramer and Mota (2015)	104	1	Advantage
Lee and Chan (2000)	85	1	Disadvantage
Ljungberg et al. (2013)	178	1	Advantage
Luk (2008) Study 1	157	1	Disadvantage
Luk (2008) Study 3	157	1	Disadvantage
Luk (2008) Study 3	157	1	Advantage
Luk et al. (2011)	123	1	Advantage
Luo et al. (2010)	60	1	Disadvantage
Luo et al. (2010)	60	1	Advantage
Luo et al. (2013)	278	1	Disadvantage
Luo et al. (2013)	278	1	Advantage
Mohades et al. (2014)	51	1	Disadvantage
Mor et al. (2015)	80	1	Disadvantage
Moradzadeh et al. (2015)	153	1	Disadvantage
Morales et al. (2013) Study 2	68	1	Disadvantage
Morales et al. (2013) Study 2	68	1	Advantage
Paap and Sawi (2014)	120	1	Disadvantage
Pelham and Abrams (2014)	90	1	Disadvantage
Pelham and Abrams (2014)	90	1	Advantage
Pelham (2012)	90	1	Disadvantage
Pelham (2012)	90	1	Advantage
Poarch and Bialystok (2015)	120	1	Advantage
Portocarrero et al. (2007)	78	1	Disadvantage
Prior and Gollan (2011)	131	1	Disadvantage
Prior and Gollan (2011)	131	1	Advantage
Prior and Gollan (2013)	175	1	Disadvantage
Prior and Gollan (2013)	175	1	Advantage
Prior and MacWhinney (2010)	88	1	Disadvantage
Prior and MacWhinney (2010)	88	1	Advantage
Rainey et al. (2016)	92	1	Advantage
Ransdell et al. (2006)	106	1	Advantage
Ratiu and Azuma (2015)	105	1	Disadvantage
Rietbergen (2014)	60	1	Disadvantage
Rietbergen (2014)	60	1	Advantage
Romano (2009)	81	1	Advantage
Ross and Melinger (2017) Study 1	147	1	Disadvantage
Ross and Melinger (2017) Study 1	147	1	Advantage
Ross and Melinger (2017) Study 2	90	− 1	Null
Rosselli et al. (2002)	122	− 1	Null
Rosselli et al. (2016)	114	− 1	Null
Rutkoski Rodrigues and Zimmer (2015)	78	1	Disadvantage
Rutkoski Rodrigues and Zimmer (2015)	78	1	Advantage
Ryskin et al. (2014) Exp. 1	64	1	Disadvantage
Salvatierra and Rosselli (2010)	233	1	Disadvantage
Salvatierra and Rosselli (2010)	233	1	Advantage
Scaltritti et al. (2017)	97	− 1	Null
Schroeder et al. (2016)	218	1	Advantage
Shulley and Shake (2016)	104	1	Disadvantage
Soveri et al. (2011)	65	1	Disadvantage
Soveri et al. (2011)	65	1	Advantage
Suárez (2013)	89	1	Advantage
Taler et al. (2013)	70	1	Disadvantage
Taler et al. (2013)	70	1	Advantage
Tao et al. (2011)	100	1	Advantage
Teubner-Rhodes (2014) Exp. 2	66	1	Disadvantage
Teubner-Rhodes (2014) Exp. 2	66	1	Advantage
Teubner-Rhodes (2014) Exp. 4	110	1	Advantage
Vega-Mendoza et al. (2015) Exp. 1	51	1	Advantage
Vega-Mendoza et al. (2015) Exp. 2	115	1	Disadvantage
Vinerte and Sabourin (2015)	65	− 1	Null
Vivas et al. (2017)	90	1	Disadvantage
Wierzbicki (2014)	123	1	Advantage
Wodniecka et al. (2010) Study 1	83	1	Disadvantage
Wodniecka et al. (2010) Study 2	93	1	Advantage
Woumans et al. (2015)	123	1	Advantage
Xie and Dong (2017)	126	1	Advantage
Yamasaki and Prat (2014)	260	1	Disadvantage
Yang and Yang (2016)	102	1	Disadvantage
Yang and Yang (2016)	102	1	Advantage
Yudes et al. (2011) Exp. 1	48	− 1	Null
Yudes et al. (2011) Exp. 2	48	− 1	Null

**Figure 5:**
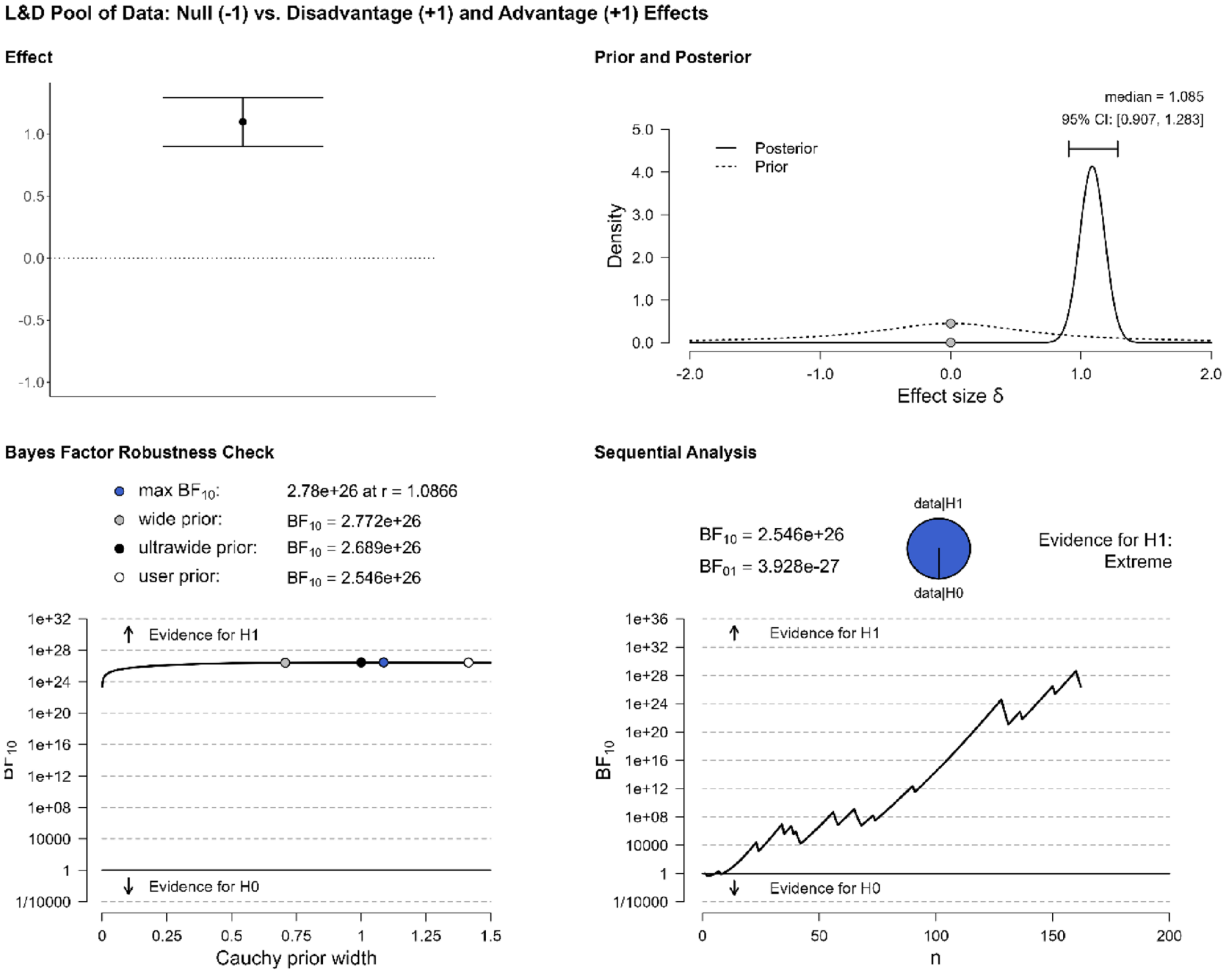
Evidence for bilingual adaptations in the L&D pool of data.

The first analysis addresses the question of whether the L&D pool of data provides robust evidence for bilingual adaptations (advantages/disadvantages vs. null). A Bayesian one-sample t-test suggests that the answer is positive, BF_10_ = 2.546e+26. Figure 5 shows that the evidence in favor of the alternative hypothesis is classified as extreme, replicating the result we obtained based on our first pool of data.

**Figure 6:**
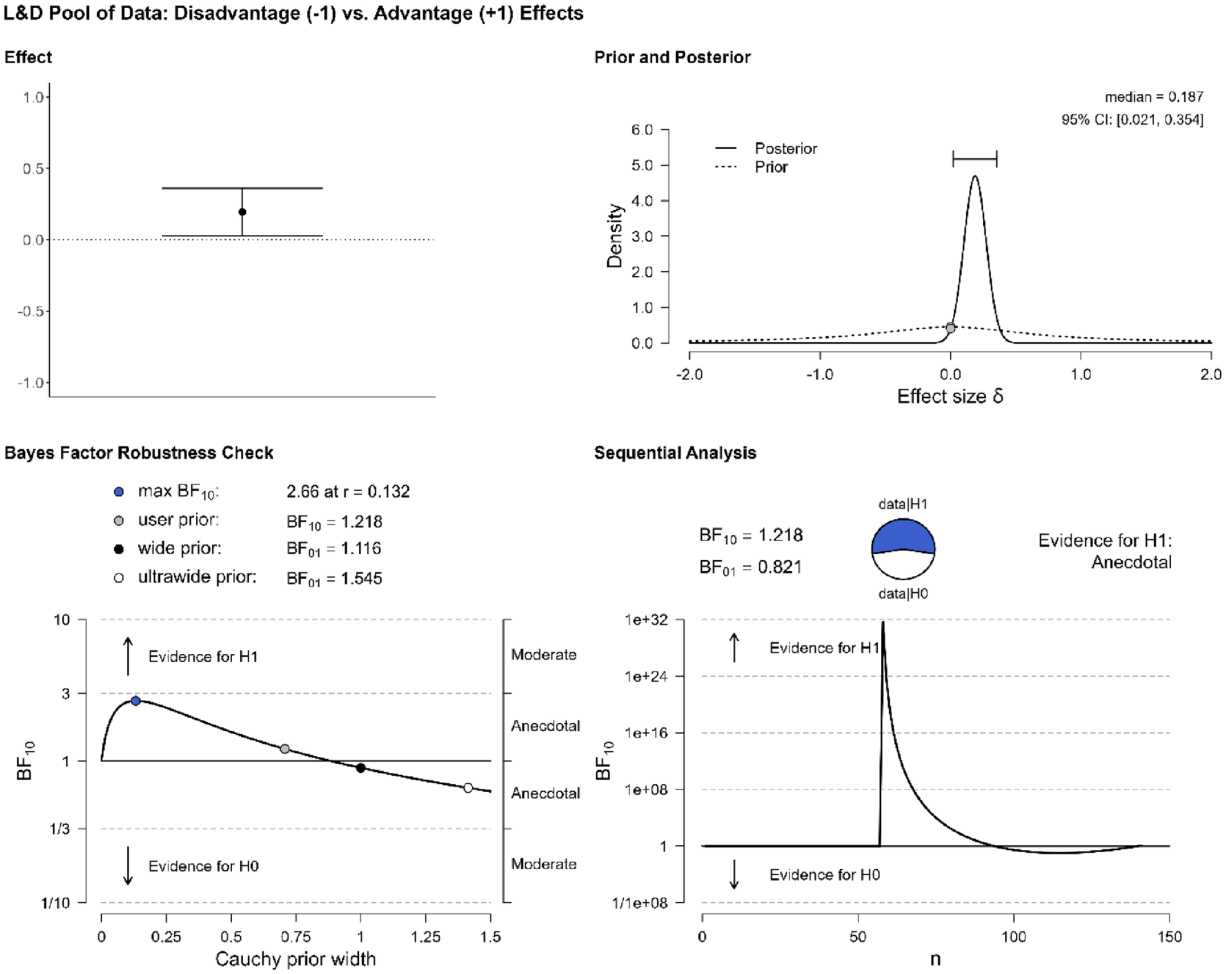
Evidence for the type of effect (advantage vs. disadvantage) in the L&D pool of data.

The second analysis concerns the occurrence of the advantages and disadvantages in the L&D pool of data. Focusing on the studies of Table 2 that find evidence for such effects (n = 93), a Bayesian one-sample t-test suggests that there is no evidence that the data are more probable under the null hypothesis vs. the alternative, BF_10_ = 1.218 (Fig. 6). This result differs from that of Grundy^19^, but the difference is explained by the fact that, unlike Grundy, we included in the L&D pool of data verbal processing tasks, and trimmed studies that involved very small sample sizes (< 30). Recall that BFs between 0.33 and 3 are considered as anecdotal or inconclusive, not offering robust evidence for either hypothesis^40^. In other words, we obtain the same result that we got in the context of the first pool of data: the data are equally likely under the null or the alternative hypothesis and we cannot sustain the claim that either advantages or disadvantages are more prevalent in our pool of data. Again, adding in this analysis the studies that find a null result (n = 12) does not alter this outcome, BF_10_ = 1.141. To address the issue of effect sizes, in the analysis of the first pool of data we followed the weighting procedure of Grundy^19^: Each study was assigned a weight by dividing the study’s sample size by the total sample size of all studies, and this proportional weight was multiplied by the effect score (i.e., − 1 for a bilingual disadvantage, 1 for a bilingual advantage) to yield a weighted score. Applying this approach in the L&D pool of data, a Bayesian one-sample t-test showed moderate evidence in favor of the data being more likely under the null hypothesis (i.e., that neither effect is more prevalent than the other), BF_10_ = 0.256, confirming the result that we got based on the first pool of data.

## Discussion

This systematic review and the two quantitative analyses investigate a question that so far lacks a clear answer: To what extent do bilingual advantages co-occur with bilingual disadvantages? Through analyzing the results of 39 studies, obtained through the PRISMA protocol, we determined the co-occurrence of positive and negative outcomes across tasks and populations. Our key finding is that, null results aside, 100% of the populations that are featured in our pool of data can be linked to both advantages and disadvantages. Specifically, in relation to the studies that reported only a disadvantage, we found at least one other study that presented a counterbalancing advantage for the same population/language group, although the caveat of comparability exists, and in some cases, we were not able to obtain a perfect match. Our two quantitative analyses, which are methodologically novel in representing all outcomes within a study, suggest that there is ample evidence for bilingual adaptations in two different pools of data (one obtained through the PRISMA method and one consisting of the datasets of two recent meta-analyses^18,36^), but no evidence that the data is more likely under the alternative hypothesis that assumes a difference in the prevalence of advantages vs. disadvantages. This means that the published record in bilingualism research features an approximately equal number of advantages and disadvantages. This finding has important implications for claims about a publication bias that favors positive results^17^, adding a tile to the literature that challenges such claims^19,20^.

We propose that this equilibrium is not accidental. Contrary to what seems to be the tacit assumption in the relevant literature, and even explicitly argued in some of the studies we analyzed (cf. the claim in^28^ on how the idea that whatever mechanism causes a bilingual disadvantage may also be responsible for a bilingual advantage seems counterintuitive), we argue that bilingual advantages and disadvantages need not be conceived as stand-alone outcomes in free variation. Instead, they could be viewed as correlations that occur simultaneously in response to some environmental trigger that causes an adaptive alteration to the system. In this sense, the effects of bilingualism on cognition would not induce advantages or disadvantages per se, but trade-offs that come in different forms (e.g., speed-accuracy, accuracy-flexibility, efficiency-resistance to noise).

Assuming the existence of a cognitive trade-off means that enhancements of a function, when observed, capitalize on resources that are used across cognitive domains, such that some aspects of performance, efficiency, robustness, or flexibility must eventually be taxed^21^. One well-established cognitive trade-off, for instance, is the speed-accuracy one for which, succinctly put, faster performance taxes responses’ accuracy^41^. This trade-off hypothesis can be conceived as consisting of different ramifications that respond to an environmental trigger (in this case, bilingualism) through playing a *zero-sum game*^42^: Once we zoom out of individual studies and observe the overall picture in a given population, positive and negative effects (i.e., bilingual advantages and disadvantages) will likely be found to complement each other, striking a balance that optimizes the use of the organism’s finite resources. Such trade-offs are inherent to cognition and life in general^21^, and there is ample evidence of applications of the trade-off hypothesis in different branches of neurocognition or evolutionary ecology^42^. Generally, trade-offs boil down to key, high-level properties of goal-directed systems (e.g., general or task-specific aspects of performance, resilience, efficiency, robustness, and flexibility). However, we still do not know what kind of trade-offs bilingualism confers and what the picture of bilingual adaptations would look like when systematically analyzed in terms of trade-offs. While certain general patterns can be discerned (e.g., the trade-off between enhanced cognitive control measures such as switching and hampered verbal fluency), the field lacks concrete answers as to what makes these two domains good candidates to pair together in a trade-off. Future research in this direction is likely to move us further away from dichotomous “advantage/disadvantage” labels, providing the relevant answers.

The existence of bilingual trade-offs does not directly follow by mere observation of the simultaneous presence of effects that go to opposite directions. Instead, it requires the *linking* of the two under a shared origin. Most studies in our pool of data do not link the two fronts, even when they adduce evidence for both advantages and disadvantages. Perhaps the most explicit exception is found in^43^ and^44^, who argue in favor of a compensatory effect, according to which a bilingual language-processing disadvantage may be offset by enhanced language-independent, sensory processes. At the same time, while the presence of both effects might be acknowledged in some studies, the general purpose of a study may grant a more prominent position to one of the two outcomes. Consequently, although many studies in our pool of data have reported both advantages and disadvantages, the overall tendency is for such results to be presented as stand-alone effects. For example, it has been suggested that the acquisition of complex grammatical phenomena is delayed in bilingual children^45^. In children with Specific Language Impairment (SLI), bilingualism has been argued to confer an additional disadvantage^46^. Following a trade-off approach, however, reduced speed in acquisition is expected to be compensated for in another domain. Indeed, our results suggest that this might be the case. Bilingual children may need more time to acquire certain grammatical phenomena, but they also demonstrate better L3 learning abilities^47^, an enhanced capacity to ignore irrelevant information^48^ and, for children with SLI, better narrative competence^49^, which may translate into overall enhanced flexibility on their part.

Our results show that bilingualism, as an environmental trigger, may confer an array of (dis)advantages that must be compensated in the opposite direction, such that it could be meaningful to talk about *cascade effects*, that consist of multiple (dis)advantages, and not about a single “advantage-disadvantage” pair with fixed components. Moreover, bilingualism is not the only activity with an impact on cognition, but one of the many, like exercising navigation skills^50^ or doing music^51,52^. In the likely event that bilingualism and other sources of adaptation co-exist in an individual^15,53^, cognitive enhancements—which imply taxing some cognitive resources—cannot infinitely add up, because of the finite resources of the system. As a result, a ceiling effect of enhancement may be reached based on one environmental trigger (e.g., music), effectively rendering other triggers (e.g., bilingual experience) null, at least for some time. For example,^51^ compare monolingual and bilingual musicians and non-musicians and find a musical training advantage on working memory, but not a bilingual advantage. However, the expectation that it is possible to observe the cumulative effect of two enhancements is perhaps too optimistic. It is likely that musicians have already reached a cognitive peak thanks to their musical advantage, such that the effect of bilingualism could not lead to further enhancements, at least not until the first source of influence wanes. Put more succinctly, if an environmental trigger has conferred a cognitive advantage that has already led to superior performance in some domain, a plateau is expected after the peak point, at least until the effect gets weaker as practice, use, and ability of the advantage-conferring experience changes over time.

There is a way in which this point relates to age. Our review of studies that tested child populations suggests that the observed effects tend to level out with time^45,54^. This fluctuation, besides being evidence for developmental plasticity^35^, can also be construed as the path towards a plateau effect: in early adulthood, performance is almost at ceiling level, such that environmental factors have less room for enhancing processes associated with cognitive control^26,55^. To exemplify, while bilingual toddlers in^54^ are invariably associated with a disadvantage in auditory word recognition, a young adult population tested for the same skill is less accurate than monolinguals, yet still faster than them at word learning, suggesting that age contributed to levelling out differences between the two groups in some domains. Similarly, the advantage of simultaneous bilingual children over early L2 learners in the acquisition of early-acquired phenomena^45^ disappears with time, and the two groups do not differ for phenomena that are typically acquired later in life.

**Figure 7:**
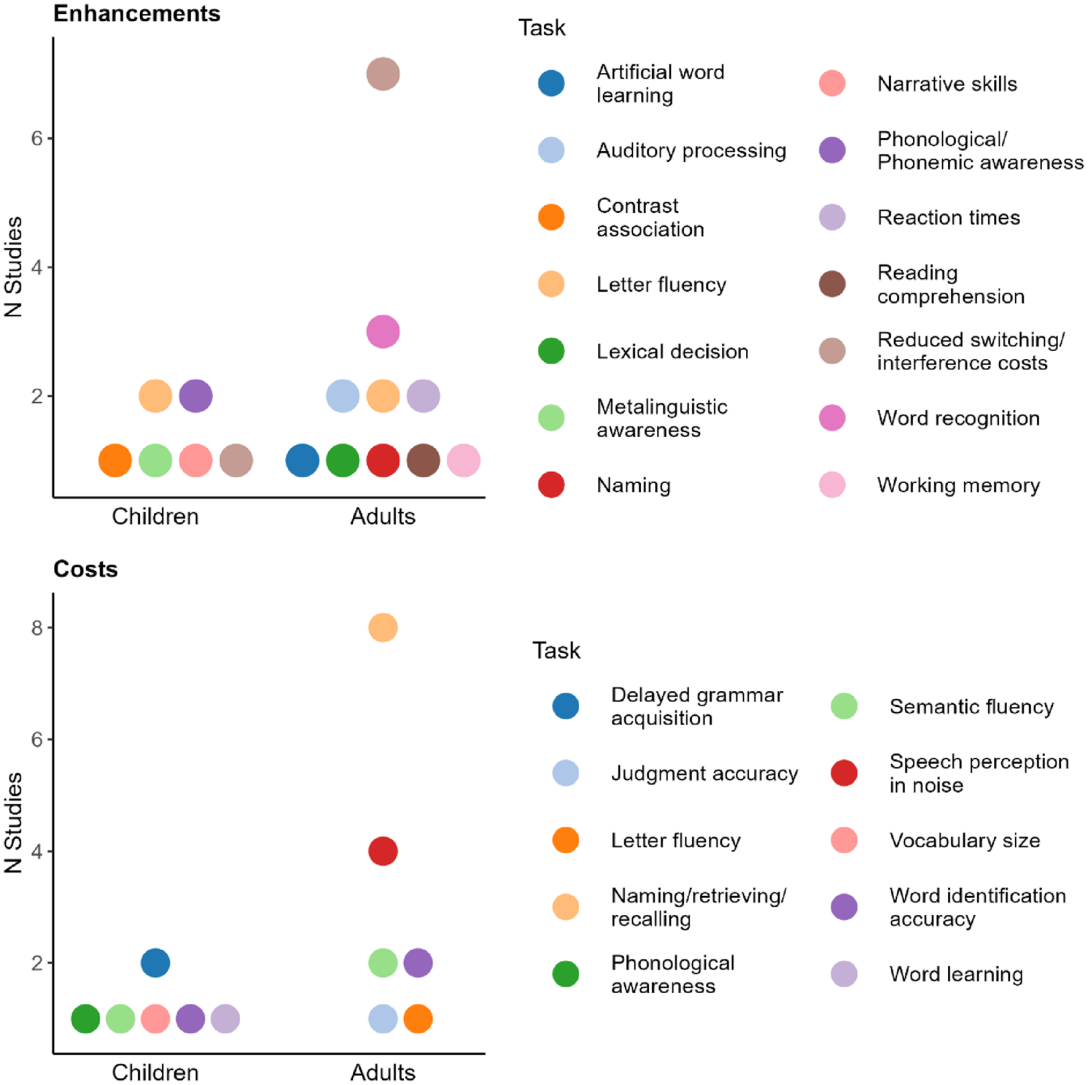
Domains that show enhancements and costs.

Overall, our results suggest that the cascade effects of cognitive adaptations to bilingualism (i) are not dichotomous, (ii) possibly form part of trade-offs, whereby enhanced performance in any domain entails certain costs and compensations, and (iii) are dynamic and subject to change under the influence of various environmental triggers, but also of time and developmental stage. Moreover, our results indicate that there is no domain that stands out in terms of showing *either* advantages *or* disadvantages. As Fig. 7 shows, certain domains of testing may be more strongly associated with either hampered or enhanced performance (e.g., semantic fluency is often associated with a bilingual disadvantage), but such relations are not absolute, such that it is possible that a domain that is frequently linked to a(n) (dis)advantage also shows results that go in the opposite direction. The considerable domain overlap that is shown in Fig. 7 illustrates this point.

Notwithstanding the outlined variability in our results, a pattern compatible with what has been noted in the literature can be observed: Bilingual disadvantages are often associated with the verbal domain, whereas enhanced bilingual performance is found in tasks involving executive function measures^56–58^. In our pool of data, the majority of bilingual disadvantages are observed primarily in language production and, to a lesser extent, in speech recognition abilities^44,54^. Conversely, the reported bilingual advantages manifest both (i) as reduced switching/interference costs in the non-verbal domain^54,59^ and (ii) in the verbal domain, as improved performance in tone/speech-in-noise recognition^44,60^, picture naming^61,62^, word recall^63^, and letter and phonemic fluency^28,64^.

Adding to advantages manifesting in tasks that test linguistic abilities, opposite bilingual effects also manifest within the *same* cognitive domain. In our database, this is exemplified by^64^ and^28^. Both studies focus on language production and while they report a letter fluency bilingual advantage over monolinguals, such enhancement is offset by a cost in category fluency. Marsh et al.^28^ attribute this finding to heightened executive processes involved in letter fluency—where processes of phonological/lexical retrieval are heavily involved—as opposed to category fluency, which is more closely associated with the access of semantic knowledge^65^. Overall, the non-trivial systematicity of the relations “verbal domain–disadvantage” and “non-verbal domain–advantage” warrants more detailed investigation of the ways in which advantages and disadvantages tie with one another.

Although our results provide evidence for robust bilingual adaptations that may be classified in terms of positive and negative outcomes, we suggest that it is not useful to employ labels like “bilingual advantage” and “bilingual disadvantage” in isolation, without situating them within a greater bundle of effects. Moreover, if we view such effects as the connatural components of a trade-off, cautionary notes about the inconsistency of the behavioral data—an inconsistency that is often used to criticize the bilingual-advantage hypothesis (see^66^ and references therein)—cease being relevant: Results might be inconsistent because they often seek to explain one of the two parts of the trade-off in isolation, as if advantages and disadvantages were stand-alone effects. Yet, they may not be, and the equilibrium we observed in terms of overall advantages and disadvantages suggests that both sides of the debate have adduced a high volume of results that reliably find evidence for a bilingual effect, either positive or negative. This equilibrium means that there is no overall bilingual cognitive (dis)advantage per se: advantages in some areas will result in disadvantages in other cognitive domains, due to the dynamic nature of the induced trade-offs. While it can be claimed that bilingualism always entails an advantage in the sense of being exposed to a different linguistic and possibly cultural reality^4^, the measurable cognitive differences subsumed under the labels “bilingual advantage” and “bilingual disadvantage” are probably temporary, as language (like any other trait) gradually transitions from a heavily controlled, mentally effortful process to a more automated one^67^.

Aiming to capture the bigger picture, it seems that an interesting parallel can be observed between brain and behavior. Under the trade-off approach outlined in the present work, the cognitive front seems to correlate with the picture observed at the neuroanatomical level. Similar to how the behavioral data do not point to a single trade-off, but to many (e.g., accuracy vs. flexibility, speed vs. accuracy), it seems that there is no single, fixed locus for bilingual adaptations at the level of the brain either. While ample and reliable evidence for brain adaptations to bilingualism has been found^30,68,69^, there is no single locus for these adaptations^70^, and alterations in the connectivity or volume of various cortical regions and subcortical structures have been observed^71,72^. Overall, this variation observed at the various fronts is evidence for differential adaptations to bilingualism. This offers support to recent approaches to bilingualism as a spectrum experience that permits variable modulations based on individual language experiences^32,73,74^.

## Conclusion

Research in the topic of cognitive adaptations to bilingualism has produced largely contradictory results that can be classified into three categories: positive evidence, negative evidence, and null. While many explanations have been offered for the different sets of results, a matter that has remained unaddressed is the degree to which advantages and disadvantages coexist across tasks and populations, such that they possibly rise to a balanced bilingual effect. This systematic review and two quantitative analyses addressed this question in a novel way, through representing all the outcomes reported in the analyzed studies. First, we determined that there is strong evidence for the presence of bilingual adaptations (in the form of both advantages and disadvantages) in the analyzed datasets, indicating that bilingualism is indeed more likely than not to exert effects on cognition. Second, our quantitative analyses showed that the data support the hypothesis that positive and negative outcomes are equally plentiful. This finding is in agreement with previous meta-analyses that find more advantages than disadvantages in the executive domain^19,75,76^, because—under the trade-off approach we have laid out—any possible executive function advantages are likely offset in other cognitive domains, including possible disadvantages in verbal processing tasks.

We have argued that this equilibrium may not be accidental. The distribution of effects suggests that the terms “bilingual advantage” and “bilingual disadvantage” should not be conceived as stand-alone effects, forming a single “advantage/disadvantage” pair, but possibly as inseparable parts of an overall trade-off that comes in different guises and forms (e.g., accuracy vs. flexibility of switching, accuracy vs. speed, affected lexical retrieval vs. enhanced pragmatic monitoring, etc.). From this perspective, advantages in some cognitive measures will result in disadvantages in others, playing a zero-sum game that can be best explained through assuming dynamic trade-offs. The effects subsumed under the labels “advantage” and “disadvantage” offset each other in a way that makes them subject to change under the influence of environmental factors such as time and developmental stage. To conclude, while reasoning in fixed terms may be convenient, not recognizing the composite nature of both bilingualism and its effects would pose an unnecessary limit to our understanding of bilingual cognition. It is likely that future research on the topic will address the remaining open questions (cf.^33^) while controlling for research practices so as to reflect the diversity of bilingualism (cf.^77^), and develop a theory of the characteristics of the bilingual trade-offs, hopefully as part of an overall theory of cognitive adaptations to bilingualism that addresses the factors that drive the observed effects.

### Supplementary Information

Supplementary Tables.

## Data Availability

All data associated to the current study are available in this published article and in the OSF project, https://osf.io/8w6ux/.
