# Supplementary material for: Bilingual disadvantages are systematically compensated by bilingual advantages across tasks and populations
Source: Sci Rep. 2024 Jan 24;14:2107. doi: 10.1038/s41598-024-52417-5 (PMC10808122; doi:10.1038/s41598-024-52417-5)
Supplement: Supplementary file 1 — Supplementary Tables. [file 41598_2024_52417_MOESM1_ESM.docx]

**Bilingual Disadvantages are Systematically Compensated by Bilingual Advantages Across Tasks and Populations**

Vittoria Dentella, Camilla Masullo, and Evelina Leivada

**Supplementary materials**

**Contents:**

1. Supplementary Table 1
2. Supplementary Table 2
3. Details for the statistical analysis
4. References

**Supplementary Table 1: Summary of the results**

| **Category** | **%** | **No. of Studies (n=39)** |
| --- | --- | --- |
| Advantage & Disadvantage | 20.51% | 8 |
| Neither Advantage, Nor Disadvantage | 28.20% | 11 |
| Advantage | 10.25% | 4 |
| Disadvantage & External Advantage | 41.02% | 16 |
| Disadvantage | 0.00% | 0 |

**Supplementary Table 2: Demographics**

| **Category** | **Children  (n=7)** | **Young Adults (n=24)** | **Mixed-age (n=8)** |
| --- | --- | --- | --- |
| Advantage & Disadvantage | 28.57% (2) | 16.66% (4) | 25.00% (2) |
| Neither Advantage, Nor Disadvantage | 14.29% (1) | 29.16% (7) | 37.50% (3) |
| Advantage | 0.00% (0) | 12.5% (3) | 12.50% (1) |
| Disadvantage & External Advantage | 57.14% (4) | 41.66% (10) | 25.00% (2) |
| Disadvantage | 0.00% (0) | 0.00% (0) | 0.00% (0) |

**Details for the statistical analysis**

The Bayes factor (BF) computes the probability of the analyzed data under the null hypothesis vs. the alternative hypothesis [1, 2]. A BF of 10-30 is typically considered strong support for the alternative hypothesis, while a BF>100 can be interpreted as extremely strong evidence in favor of the alternative [3]. In contrast to the frequentist *p* value, the BF allows researchers to quantify the evidence in favor of the null hypothesis by determining that the results are *X* times more likely under the alternative than under the null [2, 4].

**References**

[1] Jeffreys, H. (1961). *Theory of Probability*. Oxford: UK Oxford University Press.

[2] Wetzels, R., & Wagenmakers, E. J. (2012). A default Bayesian hypothesis test for correlations and partial correlations. *Psychonomic Bulletin and Review*, 19(6), 1057–1064.

[3] Grundy, J. G. (2020). The effects of bilingualism on executive functions: An updated quantitative analysis. *Journal of Cultural Cognitive Science*, 4(2), 177-199.

[4] Dienes, Z. (2014). Using Bayes to get the most out of non-significant results. *Front. Psychol. 5*:781. doi: 10.3389/fpsyg.2014.0078
